# Addressing the Interactive Effects of Maltreatment and COVID-19 Related Stressors on the Neuropsychological Functioning in Children

**DOI:** 10.3389/fpsyg.2021.764768

**Published:** 2021-11-24

**Authors:** Natalia E. Fares-Otero, Sebastian Trautmann

**Affiliations:** Department of Psychology, Medical School Hamburg, Hamburg, Germany

**Keywords:** child abuse, domestic violence, cognitive functioning, social behavior, learning, neuropsychological rehabilitation, stress, COVID-19 outbreak

## Introduction

The novel coronavirus disease (COVID-19) has as of mid of October 2021, affected over 220 countries (with over 237,655,300 confirmed cases and 4,846,980 deaths; WHO, 2021a) and unprecedented disruption in the daily lives of people worldwide. Efforts to slow viral transmission, including quarantine and school closures (Baron et al., 2020; Engzell et al., 2021), have introduced profound changes in family routines and children circumstances. The conditions created by the COVID-19 pandemic (Bridgland et al., 2021; Calvano et al., 2021; Gadermann et al., 2021) have heightened the likelihood for both life stressors (Brown et al., 2020; Griffith, 2020; Achterberg et al., 2021; Mohler-Kuo et al., 2021; Spinelli et al., 2021) and childhood maltreatment (CM; Bérubé et al., 2020; Lawson et al., 2020; Rodriguez et al., 2021; Wong et al., 2021), while clinical and social services are weakened and resources for support are reduced (Jentsch and Schnock, 2020; Usher et al., 2020).

CM is already highly prevalent and widespread (Gilbert et al., 2009; Stoltenborgh et al., 2012; Hillis et al., 2016; UNICEF, 2021). It is estimated that one out of two children aged 2–17 years old experience some form of CM each year (Hillis et al., 2016), emotional abuse affects one in three children (Stoltenborgh et al., 2012), and one in four children lives with a mother who is the victim of intimate partner violence (UNICEF, 2021). Given the lockdowns, social restrictions and living in confinement coupled with massive economic disarray, we could expect a dangerous increase in the negative consequences of CM (Garstang et al., 2020; Pereda and Díaz-Faes, 2020), affecting those who, before COVID-19, have already suffered CM, but also putting children at risk for CM who were previously unaffected. Additionally, the protective measures for preventing the spread of the virus have heightened the risk for specific types of CM [e.g., online abuse or bullying, criminal, child sexual exploitation (Kuehn, 2020), and domestic violence (Evans et al., 2020; Pereda and Díaz-Faes, 2020; Rodriguez-Jimenez et al., 2020; UN Women, 2020; Cappa and Jijon, 2021)].

The clinical implementation of measures for the detection of CM is a priority. Even with the vaccine's anticipated impact on preventing the spread of the disease, new waves of COVID-19 pandemic are hitting many parts of the world driven by new variants of the virus (WHO, 2021b). There will be long lasting health, economic, developmental, and social impacts of COVID-19. It is plausible to believe that, after COVID-19, problems will not disappear for children who will continue to suffer the consequences of this crisis. It is, therefore, critical to understand and strengthen the well-being of children with pre-existing vulnerabilities (as a history of CM), and highlight key research targets to advance our knowledge of the challenges those affected by CM and stressful events (Bridgland et al., 2021) during COVID-19 are facing (Fares-Otero et al., 2020, 2021).

As a chronic stressor (Harkness et al., 2006; Wade et al., 2019; Rousson et al., 2020), CM contributes to alterations in the development and functioning of the brain (Teicher et al., 2012, 2016; De Brito et al., 2013; Mueller and Tronick, 2019), linked to neuropsychological deficits (Wilson et al., 2011; Samuelson et al., 2012; Spann et al., 2012; Blair et al., 2019), which lead to poor daily living skills (Kavanaugh and Holler, 2015; Meng et al., 2019), academic (Perez and Widom, 1994) and social maladjustment in children (Shonk and Cicchetti, 2001; Veltman and Browne, 2001; Stirling et al., 2008; Jaffee and Maikovich-Fong, 2011). Here, we propose that children exposed to CM and COVID-19 related stressors (Bridgland et al., 2021), would be more vulnerable to aggravation of neuropsychological deficits and thus, they would have higher likelihood for cognitive and social impairment through these (neuropsychological) deficits (than children without CM exposure). Therefore, it is important to identify and target neuropsychological alterations related to CM in the context of COVID-19.

## Open Questions

Although there is strong theoretical and empirical evidence for interactive effects of CM and conditions related to the COVID-19 pandemic, several important open questions remain. These include: (1) The potential dose-response relation underlying the accumulation of experienced stress types and neuropsychological risk factors during COVID-19 pandemic; (2) The interplay between effects of CM and COVID-19 stressors, examining independent contributions of co-occurring chronic and acute stress, that may contribute to the onset and maintenance of neuropsychological problems in children; and (3) Moderators of these associations including COVID-19 stressor (interpersonal violence vs. other as loss of a loved one, parental divorce) and CM type (deprivation vs. threat; Johnson et al., 2021), severity of experiences (Ouellet-Morin et al., 2019), age at onset, frequency and timing of CM (English et al., 2005) and COVID-19 stressor, gender (Sternberg et al., 2006), prior experience of CM (Guo et al., 2020), and early adverse care histories vs. current adversity, might be considered.

## A Tailored Neuropsychological Rehabilitation Program

**Figure 1:**
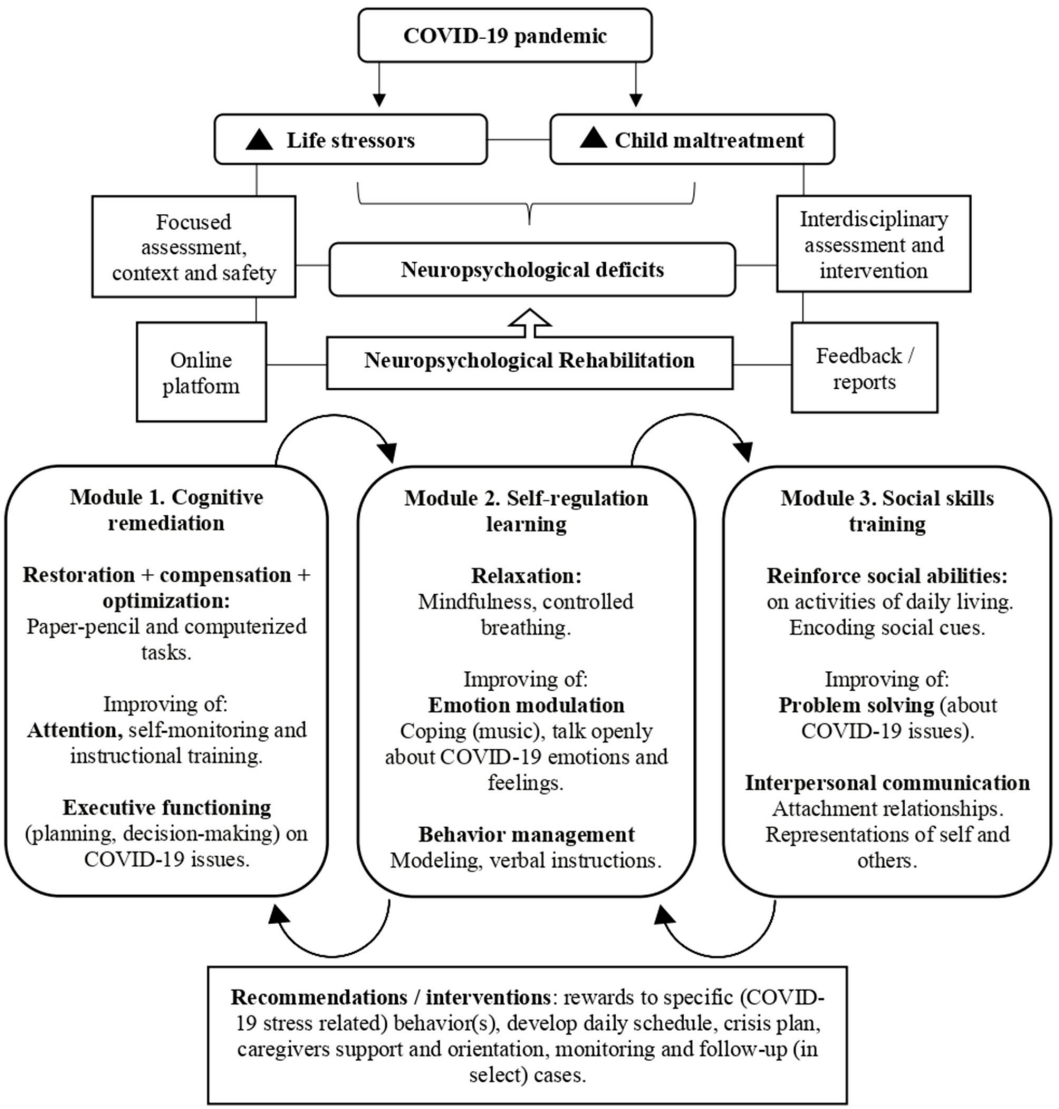
Principles and activity modules of the neuropsychological rehabilitation program for children affected by maltreatment in the context of COVID-19 stressors.

There is a need for specific interventions targeting neuropsychological difficulties in CM victims (Nolin and Ethier, 2007; Pechtel and Pizzagalli, 2011; Wilson et al., 2011). It is, therefore, a critical time window for the development of novel interventions for children with CM, in order to reduce the burden and costs associated with cumulative adverse stress-related consequences of COVID-19. Neuropsychological rehabilitation (NR) is a theoretical framework and integrated approach (Wilson, 2008; Yi and Belkonen, 2011) that appears highly promising in terms of reducing neuropsychological problems in children. Within this background (see also Figure 1), we propose a NR program to guide treatment, discharge planning, and explore ways to combat neuropsychological problems tailored to the needs of children affected by CM and COVID-19 stressors.

The proposed NR program makes use of three mechanisms: (1) The child neuroplasticity allowing the brain to be malleable, i.e., cerebral mechanisms of neuronal renewal and changes (Pal and Elbers, 2018; Weyandt et al., 2020); (2) The preserved cognitive functions (training preserved functions may ensure correct functioning of altered ones); and (3) The resilience capacity, allowing positive adaptation in the face of adversity and environmental stressors (Charney, 2004; Masten, 2007; Cicchetti, 2013; McEwen et al., 2014; Kalisch et al., 2017). The program makes further use of classic principles of NR: compensation, substitution, and restoration or optimization (Zangwill, 1947) and integrates bottom-up and top-down approaches (Rauss and Pourtois, 2013) in three modules each of which is focused on specific (cognitive, behavioral, and social) domains: 1. Cognitive remediation, 2. Self-regulation learning, and 3. Social skills training.

### Cognitive Remediation

We propose to combine (home-based) paper-and pencil (e.g., mazes, trail-making) and computerized tasks (van der Oord et al., 2014; Corti et al., 2020; Resch et al., 2021) (optionally timed and in adapted formats), with the attention training technique (by using auditory attentional exercises; Wells, 2002), self-monitoring attention and self-instructional training to engage children in executive control skills (Hallahan and Sapona, 1983; Diamond, 2012), selective and divided attention, and switching (Wells and Matthews, 1996; Ottowitz et al., 2002), and to increase top-down attentional control and flexibility, also related to delay gratification (Murray et al., 2018). Additionally, executive functioning can be trained by planning (e.g., using checklists, day planner, routines), management of time (limits) tasks, reasoning (e.g., analogies), spell out the rationale, and decision-making (on COVID-19 issues). It is important to encourage children to focus, remember and learn new information given, by using tasks of short duration to avoid fatigue and of a game format to avoid monotony, and to explore different ways of learning. An increased sense of the world being threatening in CM victims can increase child hypervigilance to (potential) threat. In addition, models of (past) threatening relationships may become activated during the COVID-19 pandemic (Kalia et al., 2020). Triggering of past traumatic memories and (possible) current ones (e.g., loss and grief) likely to be more frequent. It can be hard for children with a history of CM to place these current trauma experiences in perspective and understand that they are not permanent, and importantly, not their fault. These memories and worries could lead to attention, concentration problems and difficulty in daily planning. Children might be encouraged to manage unforeseen situations and to be flexible in order to change initial plans to adapt them to the (COVID-19) context.

### Self-Regulation Learning

We propose to train self-regulation by using relaxation techniques (Ozamiz-Etxebarria et al., 2020), infant mindfulness-(school)based interventions (Sibinga et al., 2013; Tao et al., 2021), and emotional modulation by means of music and coping behavior (Hillecke et al., 2005; von Georgi et al., 2009; Steinberg et al., 2021) to reduce COVID-19 anxiety levels, and to address stress management and behavioral difficulties (inhibitory control). The world during the COVID-19 pandemic has become a much more uncertain place. This creates anxiety for all, but particularly for children who have been reared in maltreating environments where their world in the past was also deeply uncertain. The increased wariness for bad things to happen [e.g., children may worry about contagion (Muñoz-Navarro et al., 2021) or that their caregivers become ill or lose their jobs or no longer be able to look after them] could be manifested in different kinds of feelings, particularly anxiety, but also behaviors such as irritability, aggression, and withdrawal. Thus, rules and norms (on COVID-19) should be explained to address issues of safety, stabilize impulsive aggression against self and others, and process both the traumatic memories and trauma-related expectations (Streeck-Fischer and van der Kolk, 2000). Behavioral strategies can be included as: (a) Modeling: children are given examples of situations to demonstrate them how to react or behave; (b) Reinforcement: children are given verbalizations such as “you have done well!” or “you can do it better” as positive rewards for achievements or correct execution; (c) Verbal instructions: expected behavior has to be explained directly; and (d) Dramatization: children learn behavior while acting. Children might be asked “to talk to themselves” (Meichenbaum and Goodman, 1971) to select objectives and plan before acting, using instructions such as “think before acting” or “do it more slowly” to respond in an adaptive way, to help them to observe what is happening in present time and physically respond to current demands instead of recreating the traumatic past behaviorally and emotionally (van der Kolk, 2003).

### Social Skills Training

Reinforcing social skills (in activities of daily living) can improve interpersonal communication (Cloitre et al., 2010), assertiveness, and problem solving (Tyler et al., 2021) on COVID-19 concerns. The improvement of social competence and communication involves empathy, respect of turns, verbal and non-verbal communication, by using techniques to train the performance on social skills (role-plays), to communicate and interact with each other (Ladd and Mize, 1983). The COVID-19 pandemic has had a major impact on children wider relationships. Children with CM have been losing out on the positive experiences of being with supportive people as friends or other family members. Differential sensitivity to the environment may have important implications for them in view of the deleterious consequences of CM on attachment relationships and on representations of self, caregiver and teacher. While a heightened sensitivity to social threat may be advantageous to children in the context of an adverse environment (Boyce, 2016), it can also become problematic in other situations. For instance, physically abused children are less accurate in encoding social cues, and are consequently more likely to respond aggressively to problematic social situations (Cicchetti and Valentino, 2006). The experience of CM may have meant a child was ignored and rejected, influencing their sense of self, and prior relationships with abusive adults (involving high anger exposure; Plate et al., 2019) may lead a child to believe others cannot be trusted. These vulnerabilities could be recovered through new positive experiences that encourage children to notice their strengths and weaknesses, by providing them strategies, support, and feedback (Prigatano, 1999).

## Principles of Program Implementation

### Interdisciplinary Assessment and Intervention

Interdisciplinary cooperation of child psychiatrists, pediatrists, psychologists, and social workers with experience in neuropsychological evaluation and rehabilitation is needed to address the need of CM victims in COVID-19 times. During the pandemic, children lost many sources of reward from family members, peers, and outdoor activities (Alonso-Martínez et al., 2021; de Figueiredo et al., 2021). This loose of reward may have been harder to deal with for CM victims. It is well-known that they are likely to show impairments in their reward system (Novick et al., 2018) manifested in a lack of motivation and low mood. These aspects (and maybe because of impaired attention; Boyd et al., 2019) warrant careful considerations of how to structure and deliver the proposed program.

Group sessions would allow children to practice with peers, whereas individual work promotes concentration and monitoring. Modules and tasks should be arranged based on an increasing level of difficulty, until it becomes possible for children to use the skills they would acquire (to jump to the next module). The modules should be organized to provide an order that children gradually acquire abilities that require building up from the most basic (i.e., attention) to the most demanding or complex processes (i.e., social behavior).

### Optimal Use of Technology

To complement the NR program, we propose making (online) information and (psycho)education sessions available to caregivers, educators, and clinicians (joining an online consultation platform) to rapport building and provide them with information to recognize, report and respond to CM (Kimber et al., 2020; Thomas et al., 2020), the aftereffects of CM and clarification regarding possible cognitive, behavioral and affective changes, the rationale of the NR program, and suggestions or strategies needed (at home or the classroom) in COVID-19 times.

### Focused Assessment of Problem, Context, and Safety Concerns

Besides discussing (current) neuropsychological issues, (possible) exhaustion and safety concerns might be considered. Carers of children with CM experiences always deserve particular attention, but especially so in this extraordinary period of lockdowns when caregiving in general (or cohabitation) can be extremely challenging. Within the household, parents are facing new demands with meeting basic and educational childcare needs and working remotely within in the home environment (Katz et al., 2021). We propose to review child's background, medical and developmental history, context, home environment and structure, usual and current supports. Home visiting-both in the form of physical services and remote access-should be delivered to offer children and their families appropriate professional support and to detect situations at risk for full-blown violence.

### Feedback, Recommendations, and Interventions

Feedback and turn-around report addressed to the caregivers are a need. After neuropsychological assessment, caregivers may briefly sign-off, allowing the team to debrief and develop a plan. Subsequently, caregivers sign-on to receive feedback. A (short) report should be written addressed to the caregivers (copy to referring health clinician and to the educators), formulated in lay language, with sufficiently detailed recommendations, *via* email communication (provided caregiver/parental permission). Reports might include consistent responses to specific (COVID-19 stress related) behavior(s), suggestions to adapt, simplify or develop daily activity schedule, safety assessment and crisis plan, and development of beginner communication systems. Importantly, intervention efforts are needed to strengthen positive parenting behaviors (validation, connection to resources). We recommend using emotion regulation and coping strategies (as cognitive reappraisal and self-compassion; Preuss et al., 2021) to foster parent-child relationships (Skopp et al., 2007; Austin et al., 2019) during such times of heightened stress, e.g., to respect for children feelings and opinions for COVID-19 questions.

## Discussion

Here, we proposed a novel NR program that might be helpful to reduce neuropsychological problems in children, taking into account the special needs of children affected by CM and COVID-19 stressors. Childhood is a sensitive period of neurological, cognitive, social, and emotional development, during which a neuropsychological intervention can make an important difference and shift the balance between risk and protective factors (Chinitz et al., 2017).

Current research on psychosocial impacts of the COVID-19 crisis has neither addressed the need for assessment and intervention targeting the neuropsychological functioning in children affected by CM. In our opinion article, we highlight the need for research aimed at a better understanding of the interactive effects of CM and COVID-19 related stressors on the neuropsychological function in children. We furthermore point out the importance of screening and monitoring CM and the necessity of the implementation of accurate evaluations of neuropsychological functioning in health care settings to prevent cognitive and social problems in maltreated children during and after COVID-19 pandemic and future crises.

A major challenge in our NR program is achieving a successful transfer to goal-attainment in real-life contexts. We believe that training neuropsychological functions as means to improve academic performance, is most promising in (maltreated) children, for whom both behavioral and domain-specific cognitive demands of formal schooling are quite novel challenges. An extra training and education for health professionals and educators on CM and its consequences on the neuropsychological functioning could contribute to the development of most adequate preventive and intervention measures and strengthen the collaboration between hospital services and schools.

Although the proposed NR program has a high theoretical foundation and a high potential to sustainably improve the neuropsychological functioning in children affected by CM in the context of the COVID-19 pandemic, many challenges regarding its implementation and empirical studies are needed to examine its feasibility, efficacy, and cost-effectiveness.

## Author Contributions

NEF-O: conceptualization, investigation, writing original draft, and writing—review and editing. ST: conceptualization, writing—review and editing, and supervision. Both authors approved the final version of the submitted manuscript.

## Funding

NEF-O was supported by the German Academic Exchange Agency DAAD: Deutscher Akademischer Austauschdienst (91629413).

## Conflict of Interest

The authors declare that the research was conducted in the absence of any commercial or financial relationships that could be construed as a potential conflict of interest.

## Publisher's Note

All claims expressed in this article are solely those of the authors and do not necessarily represent those of their affiliated organizations, or those of the publisher, the editors and the reviewers. Any product that may be evaluated in this article, or claim that may be made by its manufacturer, is not guaranteed or endorsed by the publisher.
